# SOCS-1 Mediates Ubiquitylation and Degradation of GM-CSF Receptor

**DOI:** 10.1371/journal.pone.0076370

**Published:** 2013-09-26

**Authors:** Severa Bunda, Kamya Kommaraju, Pardeep Heir, Michael Ohh

**Affiliations:** 1 Department of Laboratory Medicine and Pathobiology, University of Toronto, Toronto, Ontario, Canada; 2 Department of Biochemistry, University of Toronto, Toronto, Ontario, Canada; University of Birmingham, United Kingdom

## Abstract

Granulocyte-macrophage colony-stimulating factor (GM-CSF) and the related cytokines interleukin (IL)-3 and IL-5 regulate the production and functional activation of hematopoietic cells. GM-CSF acts on monocytes/macrophages and granulocytes, and several chronic inflammatory diseases and a number of haematological malignancies such as Juvenile myelomonocytic leukaemia (JMML) are associated with deregulated GM-CSF receptor (GMR) signaling. The downregulation of GMR downstream signaling is mediated in part by the clearance of activated GMR via the proteasome, which is dependent on the ubiquitylation of βc signaling subunit of GMR via an unknown E3 ubiquitin ligase. Here, we show that suppressor of cytokine signaling 1 (SOCS-1), best known for its ability to promote ubiquitin-mediated degradation of the non-receptor tyrosine kinase Janus kinase 2 (JAK2), also targets GMRβc for ubiquitin-mediated degradation and attenuates GM-CSF-induced downstream signaling.

## Introduction

GM-CSF and the related cytokines IL-3 and IL-5 regulate haematopoietic cell survival, proliferation, differentiation, migration, and perform effector functions such as phagocytosis or reactive oxygen species release [1]. Unlike other cytokine receptors, GMR has a significant nonredundant role in macrophage-mediated acute and chronic inflammation, pulmonary homeostasis, allergic diseases, and myeloid haematologic malignancies [2]. For example, juvenile myelomonocytic leukaemia (JMML) is an aggressive myeloproliferative neoplasm in children characterized by the over-production of monocytic cells that infiltrate the spleen, lung and liver [3], [4]. A hallmark feature of JMML is acquired hypersensitivity by clonal myeloid progenitor cells to GM-CSF. We recently demonstrated that the hypersensitivity of JMML cells harboring the most prevalent JMML-causing Cbl mutation, Y371H, to GM-CSF is due to the defective E3 ligase function of mutant Cbl(Y371H) towards Src family kinases that in turn hyper-phosphorylate and activate GMR to promote GMR hypersensitivity [5].

GMR is composed of a ligand-specific α chain (GMRα) and a β common (βc) signaling subunit, which is shared with the IL-3 and IL-5 receptors [6]. Upon binding of GM-CSF to GMRα, a higher-order signaling complex is formed that promotes the activation of non-receptor tyrosine kinases JAK2 and Src family kinases (Src and Lyn), which subsequently phosphorylate GMRβc [7]. Activated GMR serves as a docking site for adaptors and signaling molecules resulting in activation of downstream signaling [7]. While the molecular mechanisms underlying GMR activation have been extensively studied [2], negative regulation of GMR signaling has been less explored. Martinez-Moczgyzemba and colleagues previously showed that the cytoplasmic domain of βc is ubiquitylated and degraded by the proteasome in response to stimulation by GM-CSF, IL-5 and IL-3 [8]–[10]; however, the ubiquitin ligase that targets βc for ubiquitin-mediated degradation remains unknown. Here, we identify suppressor of cytokine signaling 1 (SOCS-1) as an E3 ligase that binds to and ubiquitylates βc to promote its degradation via the 26S proteasome and attenuates GMR downstream signaling.

## Materials and Methods

### Cells

HEK293 and TF-1 cells were obtained from the American Type Culture Collection. HEK293 cells were maintained in Dulbecco’s Modified Eagle’s Medium (DMEM; Wisent, St-Bruno, QC, Canada) supplemented with 10% heat-inactivated fetal bovine serum (FBS; Wisent, St-Bruno, QC, Canada) at 37°C in a humidified 5% CO2 atmosphere. TF-1 cells were maintained similarly in RPMI-1640 (Wisent, St-Bruno, QC, Canada) medium supplemented with 10% FBS and 2 ng/ml GM-CSF (Invitrogen, Burlington, ON, Canada). Stable knockdown of Cbl in TF-1 cells was generated as previously described [5].

### Antibodies

Antibodies against EPOR, GMRα, GMRβc (monoclonal and polyclonal), pERK, were obtained from Santa Cruz Biotechnology (Santa Cruz, CA, USA). Monoclonal antibodies against HA (12CA5), STAT5 and ubiquitin were obtained from Boehringer Ingelheim (Ridgefield, CT, USA), Millipore (Billerica, MA, USA), and Dako (Burlington, ON, Canada), respectively. Polyclonal antibodies against FLAG and SOCS-1 were purchased from Novus Biologicals (Oakville, ON, Canada). JAK2, pJAK2 and pSTAT5 antibodies were purchased from Cell Signaling Technology (Danvers, MA, USA). Monoclonal FLAG, β-actin and total ERK antibodies were obtained from Sigma (Oakville, ON, Canada).

### Plasmids

pSG5-GMRα and pSG5-GMRβc constructs were generously provided by Dr. Timothy R. Hercus. HA-ubiquitin plasmid was a gift from Dr. Zhijian Chen. Plasmids encoding Flag-SOCS-1, -2 and -3, SOCS-1▵SOCSBox have been previously described [11]. The triple lysine K>R mutant of βc, (K457R, K461R, K467R) [10] was generated using the QuikChange Site-Directed Mutagenesis Kit from Invitrogen (Burlington, ON, Canada) and the following primer pair: 5′-CTGCGCAGAAGGTGGGAGGAGAGGATCCCCAACCCCAGCAGGAGC

CACCTG, 5′- CAGGTGGCTCCTGCTGGGGTTGGGGATCCTCTCCTCCCACCTTCTGCGCAG. The presence of the indicated substitution mutations was confirmed by direct DNA sequencing.

### Immunoprecipitation and immunoblotting

Immunoprecipitation and Western blotting were performed as described previously [12]. In brief, cells were lysed in EBC buffer (50 mM Tris [pH 8.0], 120 mM NaCl, 0.5% Nonidet P [NP]-40) supplemented with protease and phosphatase inhibitors. Equal amount of protein was immunoprecipitated in same volume using indicated antibodies and immobilized on protein A–Sepharose beads. Immunoprecipitates were subsequently washed four times with NETN (20 mM Tris [pH 8.0], 900 mM NaCl, 1 mM EDTA, and 0.5% NP-40), boiled in SDS-containing sample buffer for 5 min, resolved on SDS-PAGE, and immunoblotted with the indicated antibodies.

### SOCS-1 knockdown via lentivirus-shRNA

The following pGIPZ plasmids from Thermo Scientific (Billerica, MA, USA) were used to generate lentivirus for indicated shRNA-mediated knockdown: RHS4346 non-silencing control and V2LHS_23983 human SOCS1 shRNA. TF-1-shSOCS-1 and TF-1-shScr cells were generated by infection with lentivirus and selection in puromycin. The knockdown of SOCS-1 was confirmed by Western blot analysis and real-time quantitative PCR (qPCR).

### RNA purification and real-time qPCR

Total RNA was extracted using the Qiagen RNeasy extraction kit (Qiagen, Hilden, Germany) as per the provided protocol. cDNA was synthesized using random hexamer primer Thermo Scientific (Billerica, MA, USA), 5X First-Strand Buffer, 0.1 M DTT, 100 mM dNTP set, and SuperScript II Reverse Transcriptase (Invitrogen). qPCR was performed using the CFX384 Real-Time PCR Detection System from Bio-Rad, (Mississauga, Ontario, Canada) and expression levels were determined with the Bio-Rad CFX Manager 3.0 software Bio-Rad (Mississauga, Ontario, Canada). Each qPCR reaction totaled 10 uL and consisted of 1∶4 diluted cDNA, 400 nM of each primer, and 2x SYBR Green Supermix Bio-Rad (Mississauga, Ontario, Canada). Amplification conditions were as follows: 95°C (3 min); 40 cycles of 95°C (10 s), 55°C (30 s); 65°C (5 s). Values were normalized to *β-Actin* mRNA and expressed relative to shScr samples (arbitrarily set to 1.0). The primer sets used were: *β-Actin* (5′-TTCTACAATGAGCTGCGTGTG-3′ and 5′-GGGGTGTTGAAGGTCTCAAA-3′); SOCS1 (5′-CGATTACCGGCGCATCACGC-3′ and 5′-TGTCGCGCACCAGGAAGGTG-3′).

### Chemicals

MG132 was obtained from Peptides International (Louisville, Kentucky, USA).

### Statistical analyses

Unpaired two-tailed Student t test was used to compare between treatment groups and cell types. All statistical analysis was performed using GraphPad PRISM 5.0 software (La Jolla, CA, USA). Statistical significance was achieved at the confidence limit indicated.

## Results

We and others identified sporadic and germline *CBL* mutations in 10–15% of JMML patients with Y371H mutation emerging as the most common mutation that resulted in the loss of Cbl’s ubiquitin ligase function [5], [13], [14]. Intriguingly, while mutations in Cbl abrogated the ubiquitin-mediated negative regulation of GMR-associated Src kinase turnover [5], the level of GMR remained unchanged (**Fig. S1**), which suggests that in the context of GM-CSF-induced signaling, Cbl is not the E3 responsible for the purported ubiquitin-mediated degradation of βc.

**Figure 1:**
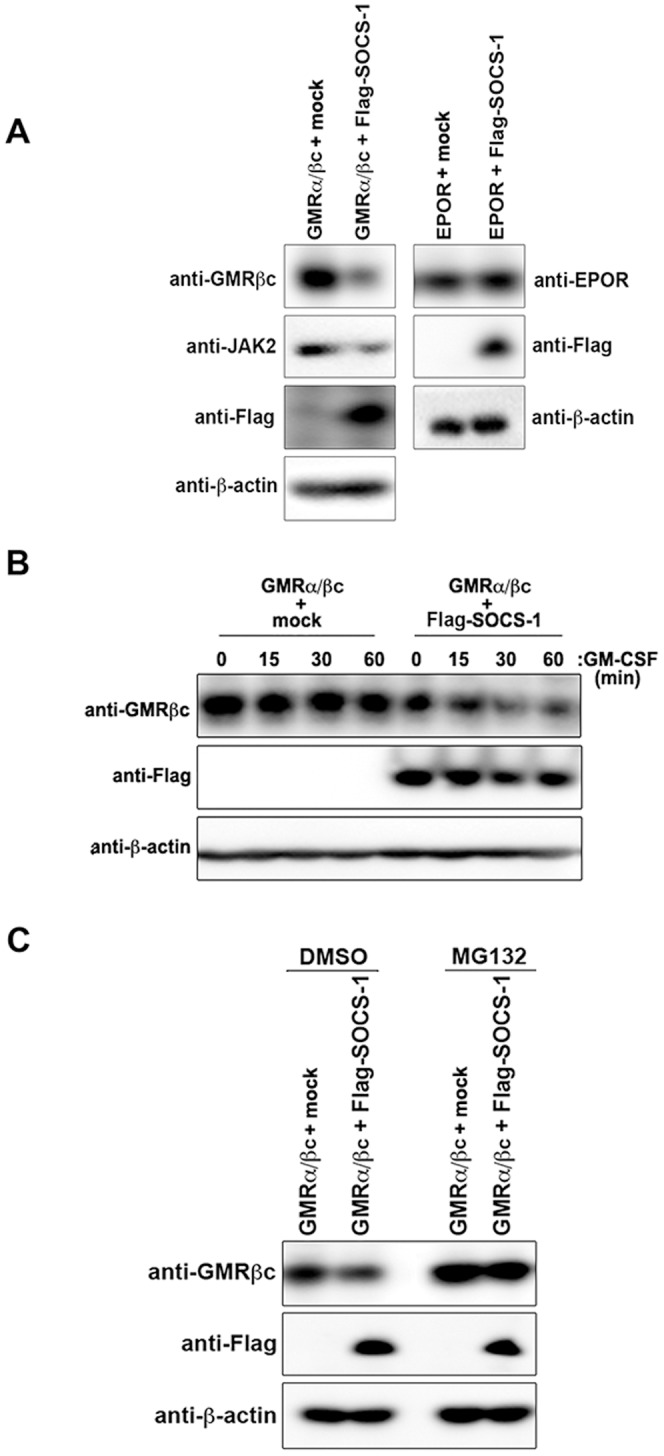
SOCS-1 decreases GMRβc expression level in a proteasome-dependent manner. (A) HEK293 cells transfected with plasmids encoding GMRα and βc (left panel) or EPOR (right panel) with or without Flag-SOCS-1 or empty plasmid (mock) were lysed and immunoblotted with the indicated antibodies. (B) HEK293 cells transfected with plasmids encoding GMRα and βc with or without Flag-SOCS-1 or empty plasmid (mock) were serum starved, stimulated with GM-CSF for indicated times and lysed for immunoblotting with the indicated antibodies. (C) HEK293 cells transfected with plasmids encoding GMRα and βc with or without Flag-SOCS-1 or empty plasmid (mock) were treated with MG132 or DMSO for 4 h prior to cell lysis and immunoblot analysis using the indicated antibodies.

Suppressor of cytokine signaling (SOCS) proteins are negative regulators of cytokine receptor signaling [15], and SOCS-1 is the substrate-conferring component of an E3 ubiquitin ligase that has been shown to target JAK2 for ubiquitin-mediated destruction [16], [17]. Considering that E3 ligases often have multiple targets and the physical association, and therefore the close proximity, of GMRβc to JAK2, we asked whether SOCS-1 ubiquitylates GMRβc. Ectopic Flag-SOCS-1 expression in HEK293 cells attenuated the expression level of endogenous JAK2 as expected and also marked decreased the level of ectopic GMRα and GMRβc (**Fig. 1a**). However, Flag-SOCS-1 had negligible influence on the expression level of another cytokine receptor, erythropoietin receptor (EPOR), known to associate with and signal via JAK2 (**Fig. 1a**). GM-CSF-induced GMRβc degradation was also dramatically increased in cells co-expressing Flag-SOCS-1 and GMRα/βc in comparison to cells ectopically expressing GMRα/βc alone (**Fig. 1b**). Furthermore, proteasomal inhibitor MG132 treatment increased the steady state level of GMRβc and importantly, blocked SOCS-1-dependent attenuation of GMRβc level (**Fig. 1c**). These results suggest that SOCS-1 promotes proteasome-dependent turnover of GMRβc.

**Figure 2:**
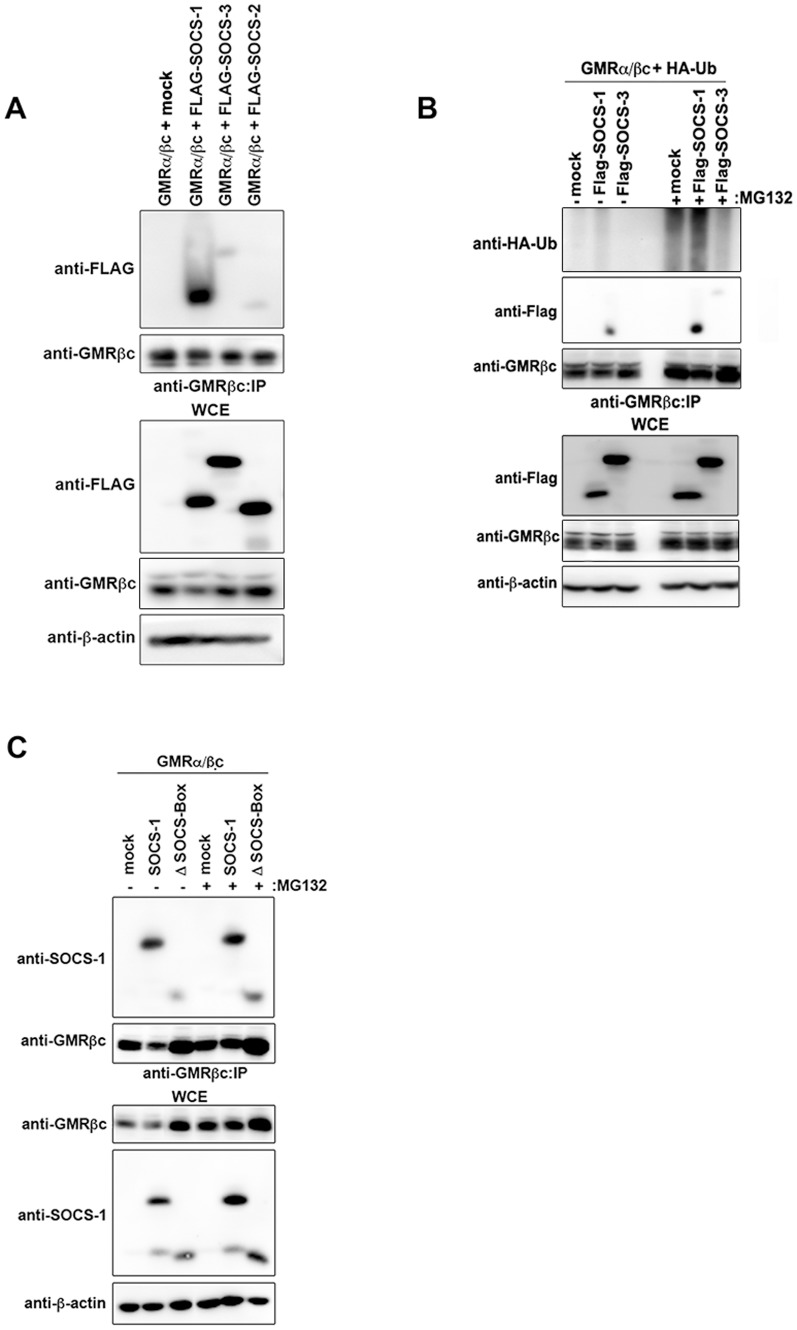
SOCS-1 preferentially binds to and ubiquitylates GMRβc. (A) HEK293 cells transfected with plasmids encoding GMRα and βc in combination with Flag-SOCS-1, -2, -3 or an empty plasmid (mock) were lysed, immunoprecipitated (IP) using anti-GMRβc antibody, and immunoblotted with the indicated antibodies. (B) HEK293 cells transfected with plasmids encoding GMRα, βc and HA-ubiquitin (HA-Ub) in combination with Flag-SOCS-1, -3 or an empty plasmid (mock) were treated for 4 h with (+) MG132 or (−) DMSO then lysed, immunoprecipiated (IP) using anti-GMRβc antibody, and immunoblotted with the indicated antibodies. (C) HEK293 cells transfected with plasmids encoding GMRα and βc in combination with Flag-SOCS-1, -SOCS-1▵SOCS-Box mutant, or empty plasmid (mock) were treated for 4 h with (+) MG132 or (–) DMSO then lysed, immunoprecipiated (IP) using anti-GMRβc antibody, and immunoblotted with the indicated antibodies. WCE: whole cell extract.

We next asked whether SOCS-1 associates with GMRβc. Flag-SOCS-1 preferentially co-precipitated with GMRβc whereas GMRβc co-precipitated minimal or negligible levels of the closely related family members SOCS-2 or SOCS-3 (**Fig. 2a and b**). In addition, SOCS-1, but not SOCS-3, promoted GMRβc ubiquitylation, which was visualized upon treatment with MG132 (**Fig. 2b**). The SOCS-box domain within SOCS-1 is required to recruit elongins B/C and Cul5 or Cul2 to form an E3 ubiquitin ligase complex and is indispensable for mediating the ubiquitylation of target proteins [18]. The SOCS-box deletion mutant of SOCS-1, while retaining modest binding to GMRβc, increased the total level of GMRβc, which was most noticeable in the absence of MG132 (**Fig. 2c**). These results suggest that SOCS-1 binds to and ubiquitylates GMRβc.

**Figure 3:**
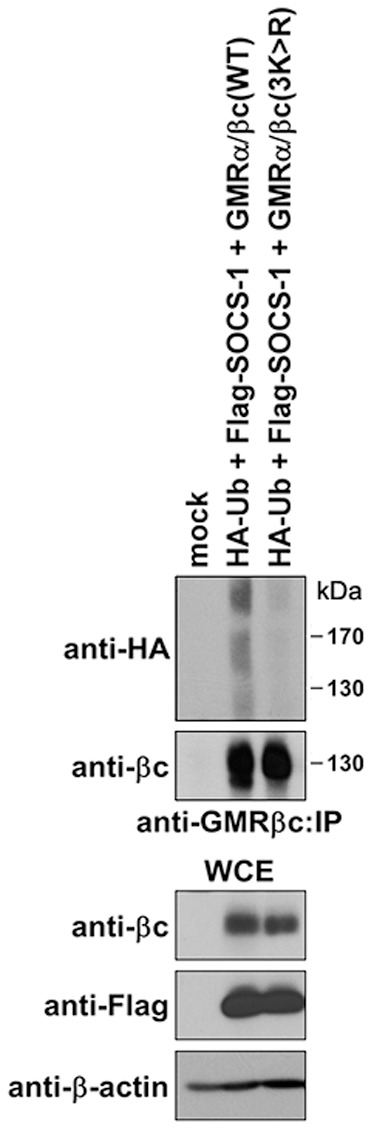
SOCS-1 ubiquitylates GMRβc on K457, K461 and/or K467. HEK293 cells transfected with plasmids encoding GMRα, βc (WT or 3K>R mutant) and HA-ubiquitin (HA-Ub) or empty plasmid (mock) were treated with GM-CSF and MG132 then lysed, immunoprecipitated (IP) using anti-GMRβc antibody, and immunoblotted with the indicated antibodies. WCE: whole cell extract.

Covalent modification of target proteins with ubiquitin occurs almost invariably on lysine residues. Previous studies have identified a cluster of three membrane-proximal GMRβc lysine residues (K457, K461 and K467) required for receptor-mediated ubiquitylation [10]. SOCS-1 mediated GMRβc ubiquitylation, which is stabilized and visualized in the presence of MG132, was markedly attenuated in GMRβc(3K>R) substitution mutant (**Fig. 3**). These results suggest that ectopic SOCS-1 promotes GMRβc ubiquitylation on one or more of these three lysine residues.

**Figure 4:**
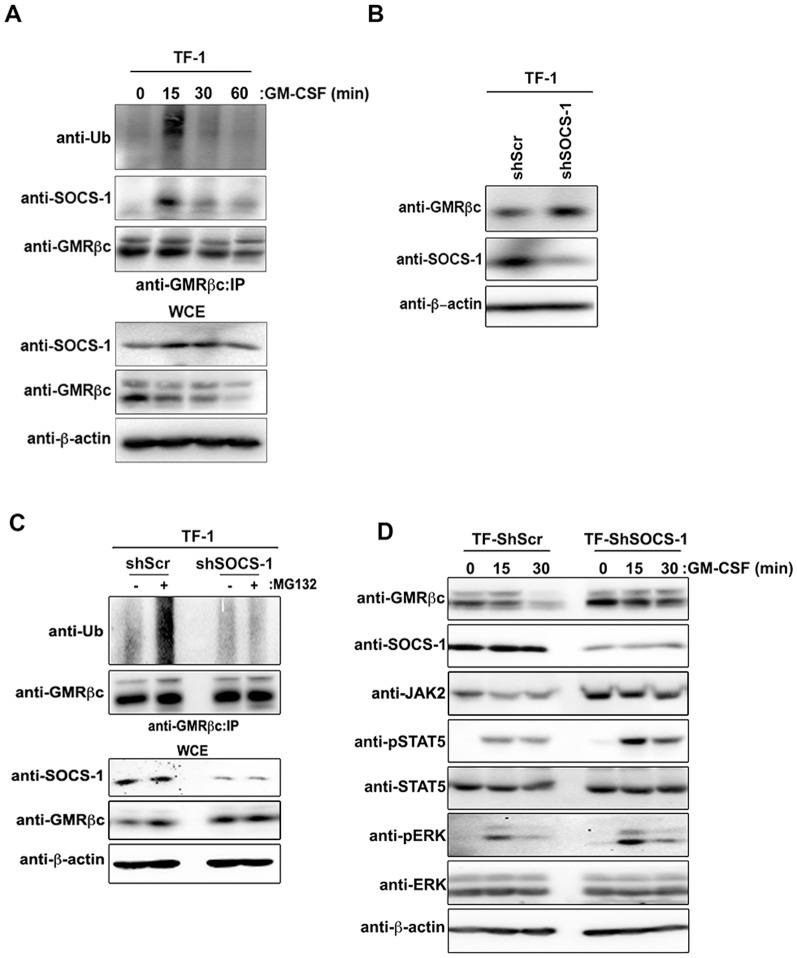
Knockdown of endogenous SOCS-1 in TF-1 cells promotes GMRβc stabilization and GM-CSF-induced downstream signaling. (A) Serum and cytokine starved TF-1 cells were treated with GM-CSF for the indicated times, lysed, immunoprecipitated (IP) using anti-GMRβc antibody, and immunoblotted with the indicated antibodies. (B) TF-1 cells transduced with lentivirus-shSOCS-1 or non-targeting scrambled shRNA (shScr) were lysed and immunoblotted with the indicated antibodies. (C) TF-1-shSOCS-1 and TF-1-shScr cells were treated with (+) MG132 or (–) DMSO for 4 h prior to immunoprecipitation (IP) with anti-GMRβc antibody and subsequent immunoblot analysis using the indicated antibodies. (D) Serum and cytokine starved TF-1-ShScr or TF-1-ShSOCS-1 cells were treated with GM-CSF for the indicated times, lysed and immunoblotted with the indicated antibodies. WCE: whole cell extract.

The human myeloid leukemia cell line TF-1 expresses endogenous SOCS-1 and GMR, and is dependent on GM-CSF for growth and survival. GM-CSF engagement of GMRα initiates rapid GMRβc recruitment and the activation of downstream signaling, which is subsequently followed by an equally rapid downregulation of GMR signaling. Upon GM-CSF stimulation of TF-1 cells, GMRβc co-precipitated with endogenous SOCS-1; the interaction of which was associated with increased GMRβc ubiquitylation (**Fig. 4a**). Moreover, the extent of GMRβc ubiquitylation and interaction with SOCS-1 diminished markedly following the initial induction upon GM-CSF treatment (**Fig. 4a**). Knockdown of endogenous SOCS-1 using lentivirus-driven shRNA against SOCS-1 in comparison to scrambled lenti-shRNA (shScr) in TF-1 cells increased the level of GMRβc (**Fig. 4b** and **Fig. S2**). The knockdown of SOCS-1 also attenuated GMRβc ubiquitylation as visualized in the presence of MG132 following GMRβc immunoprecipitation under non-saturating anti-GMRβc antibody concentration to approximate equal loading of GMRβc in the Western blot analysis (**Fig. 4c**). Moreover, GM-CSF-dependent signaling was concordantly enhanced in TF-1-ShSOCS-1 cells compared to TF-1-ShScr cells as noted by increased level and duration of phosphorylated STAT5 or ERK (**Fig. 4d**). These results support the notion that SOCS-1 promotes ubiquitin-mediated degradation of GMRβc and the negative regulation of GMR signaling.

## Discussion

The binding of GM-CSF to the ligand specific GMRα promotes the formation of a higher-order signaling complex that leads to the activation of non-receptor tyrosine kinases JAK2 and Src family kinases (c-Src and Lyn), which subsequently phosphorylate GMRβc to initiate downstream signaling [7]. SOCS-1 has been shown to negatively regulate JAK2 and consequently GM-CSF signaling by inhibiting phosphorylation [19] as well as promoting ubiquitin-mediated proteasome-dependent degradation [16] of JAK2. Here, we show that SOCS-1 also ubiquitylates GMRβc for subsequent destruction via the proteasome.

EPO-mediated signaling induces the expression of SOCS-1 [20], which is required for ubiquitin-mediated destruction of JAK2 and the suppression of EPOR downstream signaling. However, unlike GMRβc, SOCS-1 did not negatively regulate the expression level of EPOR. This is consistent with previous findings showing that JAK2-mediated degradation of EPOR is dependent on another E3 ligase containing the F-box protein β-TrCP [21],[22] and that progenitor cells from *SOCS-1* −/− mice are hypersensitive towards GM-CSF but not to other cytokines such as M-CSF or G-CSF [23], [24]. It is at present unclear why the recruitment of SOCS-1 to certain activated cytokine receptors leads to the degradation of only JAK2 via SOCS-1 while the receptor itself is targeted by another E3 ubiquitin ligase, such as in the case for the EPOR, whereas SOCS-1 recruitment to activated GMR results in the degradation of both JAK2 and GMRβc via SOCS-1. One of many possibilities is that in the case of EPOR, the duration of other downstream signaling events are likely temporally distinct from JAK2-STAT signaling; thus, there would be distinct and separate negative regulatory processes that regulate multiple downstream signaling events emanating from EPOR. In the case of GMR, it is possible that the downstream signaling pathways are regulated at similar temporal points post receptor activation. In addition, βc is shared among IL-3, IL-5 and GM-CSF receptors and hence, it may be more efficient to have one E3 ligase, namely SOCS-1-containing E3 ligase, which is responsible for the degradation of the receptor-kinase complex upon specific ligand-induced activation. These notions and whether JAK2 plays a role in SOCS-1-mediated ubiquitylation of GMRβc remain as outstanding questions.

## Supporting Information

Figure S1 **Knockdown of endogenous Cbl in TF-1 cells stabilizes Src but does not influence the rate of GMRβc turnover post-GM-CSF stimulation.** Serum and cytokine starved TF-1-ShScr or TF-1-ShCbl cells were treated with GM-CSF for the indicated times, lysed and immunoblotted with the indicated antibodies.
(TIF)

Figure S2 **Real-time qPCR analysis of shRNA-mediated SOCS-1 knockdown in TF-1 cells.** The mRNA levels of *SOCS-1* were measured by real-time qPCR in TF-1-shSOCS-1 and TF-1-shScr cells and normalized to *β-Actin* expression level. Expression level of *SOCS-1* transcripts in TF-1-shScr cells was arbitrarily set to 1.0. Error bars represent standard deviations from three independent experiments performed in triplicates.
(TIF)
