# Supplementary figures and images for: SOCS-1 Mediates Ubiquitylation and Degradation of GM-CSF Receptor
Source: PLoS One. 2013 Sep 26;8(9):e76370. doi: 10.1371/journal.pone.0076370 (PMC3784415; doi:10.1371/journal.pone.0076370)

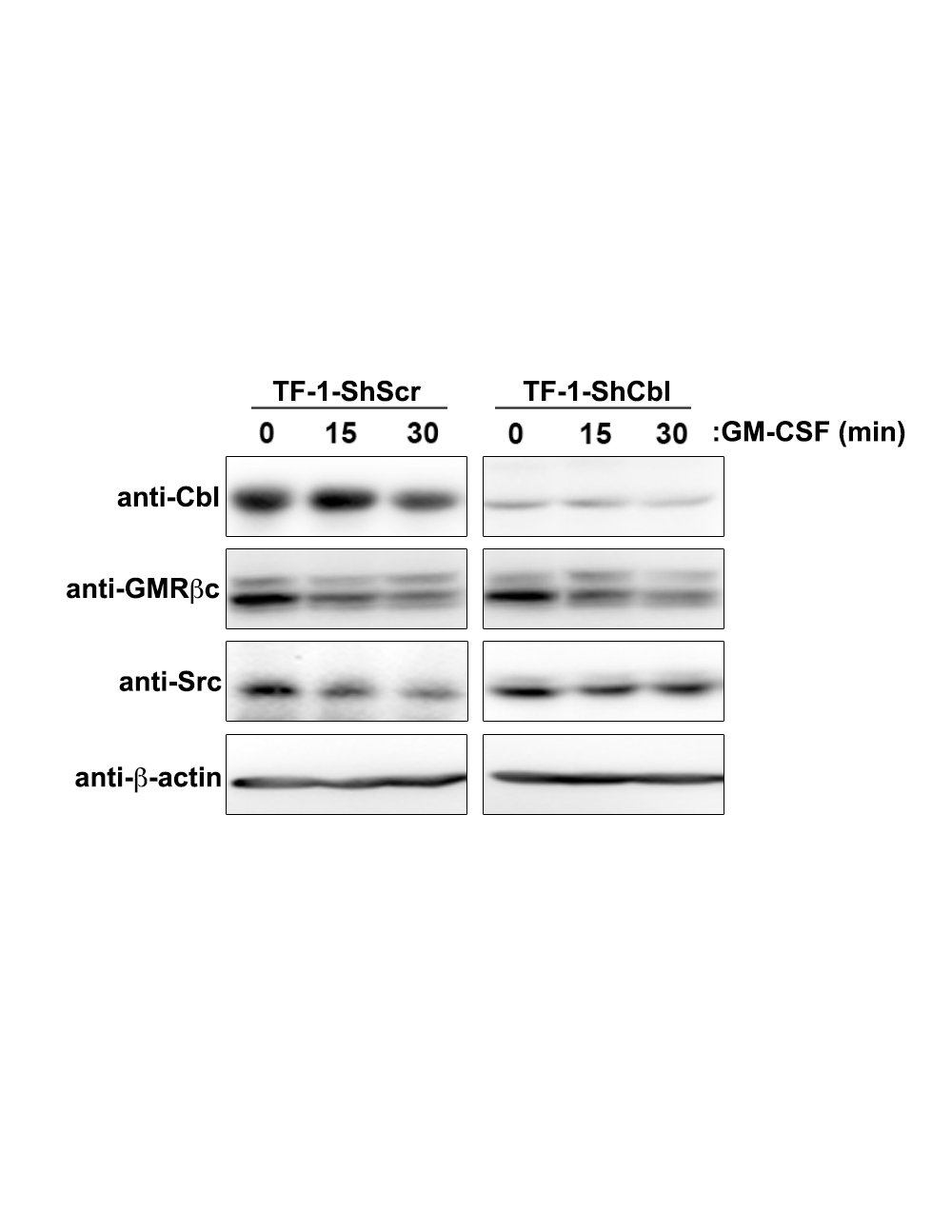

Supplement: Figure S1 — Knockdown of endogenous Cbl in TF-1 cells stabilizes Src but does not influence the rate of GMRβc turnover post-GM-CSF stimulation. Serum and cytokine starved TF-1-ShScr or TF-1-ShCbl cells were treated with GM-CSF for the indicated times, lysed and immunoblotted with the indicated antibodies. (TIF) [file pone.0076370.s001.tif]

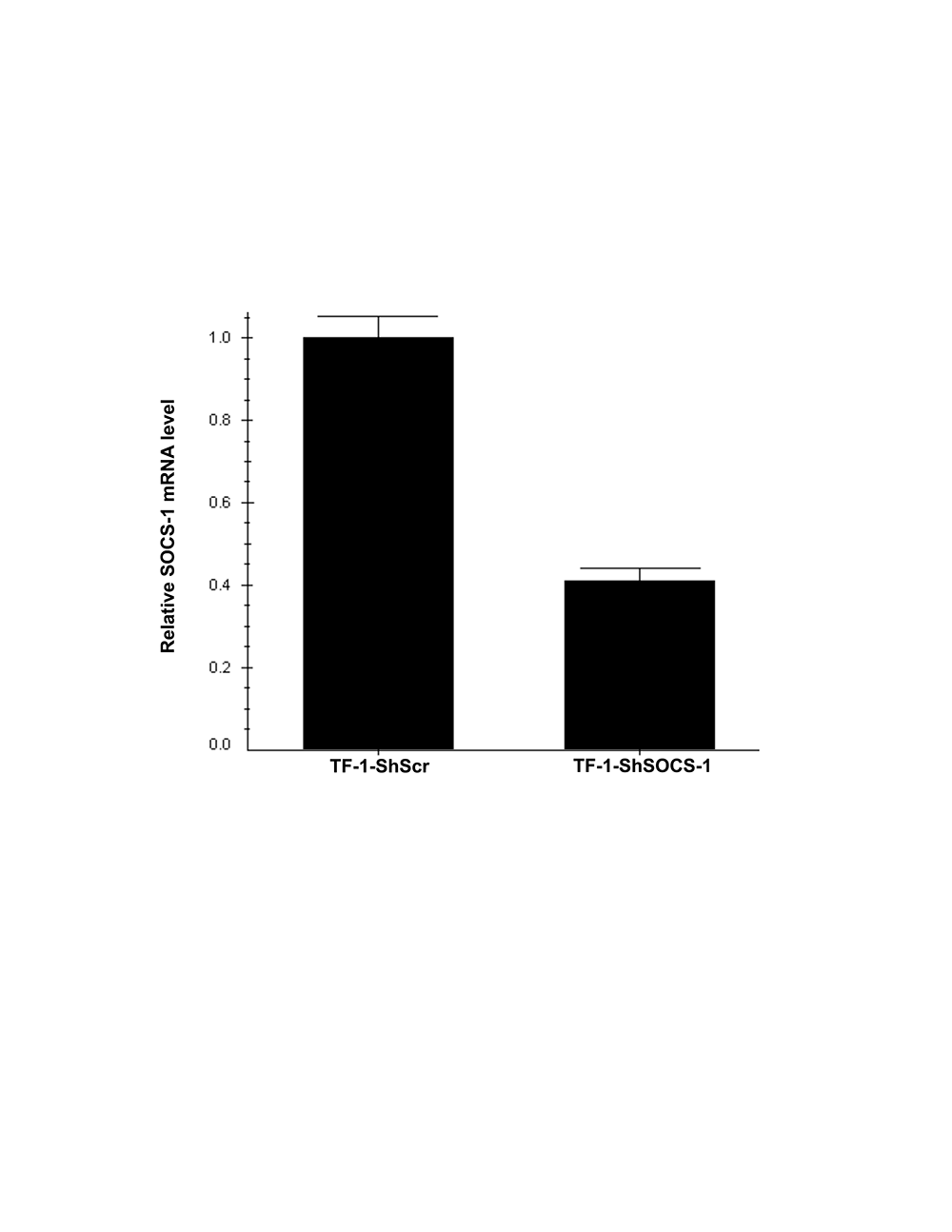

Supplement: Figure S2 — Real-time qPCR analysis of shRNA-mediated SOCS-1 knockdown in TF-1 cells. The mRNA levels of SOCS-1 were measured by real-time qPCR in TF-1-shSOCS-1 and TF-1-shScr cells and normalized to β-Actin expression level. Expression level of SOCS-1 transcripts in TF-1-shScr cells was arbitrarily set to 1.0. Error bars represent standard deviations from three independent experiments performed in triplicates. (TIF) [file pone.0076370.s002.tif]
